# Immunotherapy for the Treatment of Cutaneous Squamous Cell Carcinoma

**DOI:** 10.3389/fonc.2021.733917

**Published:** 2021-08-26

**Authors:** Andrea Boutros, Federica Cecchi, Enrica Teresa Tanda, Elena Croce, Riccardo Gili, Luca Arecco, Francesco Spagnolo, Paola Queirolo

**Affiliations:** ^1^Oncologia Medica 2, Istituto di Ricovero e Cura a Carattere Scientifico (IRCCS) Ospedale Policlinico San Martino, Genova, Italy; ^2^Department of Internal Medicine and Medical Specialties (DiMI), School of Medicine, University of Genoa, Genova, Italy; ^3^Genetics of Rare Cancers, Department of Internal Medicine and Medical Specialties, University of Genoa, Genova, Italy; ^4^U.O. Clinica di Oncologia Medica, IRCCS Ospedale Policlinico San Martino, Genova, Italy; ^5^Division of Medical Oncology for Melanoma, Sarcoma, and Rare Tumors, European Institute of Oncology (IEO), European Istituto di Ricovero e Cura a Carattere Scientifico (IRCCS), Milano, Italy

**Keywords:** immunotherapy, skin cancer, CSCC, cutaneous squamous cell carcinoma, cemiplimab, non-melanoma skin cancer, anti-PD-1 (programmed cell death-1 protein) monoclonal antibody, keratinocyte carcinomas

## Abstract

Cutaneous squamous cell carcinoma (CSCC) accounts for approximately 20% of all keratinocytic tumors. In most cases, the diagnosis and treatments are made on small, low-risk lesions. However, in about 5% of cases, CSCC may present as either locally advanced or metastatic (i.e. with locoregional lymph nodes metastases or distant localizations). Prior to the introduction of immunotherapy in clinical practice, the standard treatment of advanced CSCC was not clearly defined, and up to 60% of patients received no systemic therapy. Thanks to a strong pre-clinical rationale, clinical trials led to the FDA (Food and Drug Administration) and EMA (European Medicines Agency) registration of cemiplimab, a PD-1 inhibitor that achieved encouraging results in terms of objective response, overall survival, and quality of life. Subsequently, the anti-PD-1 pembrolizumab received the approval for the treatment of advanced CSCC by the FDA only. In this review, we will focus on the definition of advanced CSCC and on the current and future therapeutic options, with a particular regard for immunotherapy.

## Introduction

Cutaneous squamous cell carcinoma (CSCC) is a non-melanoma skin cancer of keratinocytic origin, and accounts for approximately 20% of all keratinocytic cancers, standing as the second most common neoplasm after basal cell carcinoma (BCC) (1). The main risk factors are chronic exposure to ultraviolet (UV) radiation, followed by age, fair phototype and immunosuppression [specifically related to solid organ transplantation (2), chronic lymphocytic leukemia (3), and HIV infection (4)] (5). Other risk factors like the exposure to arsenic and polyaromatic hydrocarbons can be considered occupational (6).

CSCC is characterized by a high tumor mutational burden (TMB) (7) with a large amount of UV radiation-related mutations, most notably C>T and CC>TT dinucleotide mutations (8). However, genetic mutations that could lead to a targeted treatment are infrequent, and may include PIK3CA, fibroblast growth factor receptor 3 (FGFR3), BRAF and EGFR (9).

Some hereditary syndromes may increase the risk of developing CSCC such as xeroderma pigmentosum, epidermolysis bullosa, oculocutaneous albinism, Lynch syndrome, and Fanconi syndrome (1).

Due to the heterogeneity of clinical and histologic presentations, therapeutic options, and low mortality rates, accurate data on the incidence and prevalence of CSCC are not available to-date. In Australia, where the highest incidence of skin cancer is generally recorded, there are an estimated 387 cases per 100,000 (10). In the United States, more than 700.000 new cases of CSCC are diagnosed annually, and about 3900-8800 people die each year due to this disease (11). In Europe, the incidence of CSCC ranges across different latitudes from 9 to 96 per 100.000 for male individuals and 5 to 68 per 100.000 for females (12–15).

In more than 90% of cases, the prognosis is good and treatment consists of minimally invasive surgical procedures or, in selected cases, other local therapy modalities (16). In case of primary CSCC for which curative surgery is not indicated, definitive radiotherapy (RT) may be considered as a primary treatment. Despite the lack of randomized trials comparing the outcomes of RT *versus* surgery and other local therapy modalities, in a systematic review and pooled analysis of 7 observational studies for a total of 761 primary CSCCs, the local relapse with RT was as low as 6.4% (17). However, especially in the immunocompromised patient population, in case of social difficulties, lack of caregiver support, and/or in presence of multiple comorbidities, CSCC can manifest in locally advanced or metastatic forms representing an emerging clinical problem (5). In these cases, local treatments are no longer indicated to achieve an appropriate disease control. Until few years ago, the only available therapeutic options were chemotherapy and targeted therapy (i.e., EGFR inhibitors), with poor response rates and duration of response, and frequently at the cost of unacceptable toxicities for such a frail population. With the approval by the Food And Drug Administration (FDA) and European Medicines Agency (EMA) of the anti-PD-1 cemiplimab in 2018, and of the anti-PD-1 pembrolizumab by the FDA only in 2020, immunotherapy has become the standard of care for patients with CSCC who are not eligible for curative surgery or radiotherapy (18).

In this review, we will discuss the main criteria for the identification of CSCC patients who are at high risk of relapse, and the multidisciplinary definition of locally advanced CSCC, according to the most recent guidelines. In addition to that, the results of main systemic treatment regimens will be discussed, with a focus on immunotherapy, especially regarding the key findings on the new therapeutic options and future therapeutic landscapes.

## Identification of High-Risk CSCC and Clinical Definition of Advanced CSCC

In most cases, CSCCs are detected as small or early-stage lesions that have a low risk of recurrence after an appropriate surgical treatment (16). Specifically, the overall recurrence rate has been shown in several studies to be between 2.1% and 4.6% (19). Although only few CSCC have a high risk of local or distant recurrence, it is essential to identify the high-risk patient group for a proper diagnostic and therapeutic workup, and an individualized follow-up. Risk factors can be either tumor-related (clinical or pathological) or patient-related, as indicated by the European Dermatology Forum (EDF), European Association of Dermato-Oncology (EADO), and European Organization for Research and Treatment of Cancer (EORTC) guidelines (20, 21). However, the impact of each individual risk factor is not entirely clear. In a recent meta-analysis, published data on risk factors for recurrence, metastasis, and disease-specific death of CSCC were systematically analyzed. The main results of this work were summarized in **Table 1** (22). Briefly, tumor depth was associated with the highest risk ratio of local recurrence and metastasis, while a tumor diameter > 20 mm was associated with the highest risk ratio of disease-specific death (22).

**Table 1:** Risk ratios for recurrence, metastasis, and disease-specific death for some of the most relevant high-risk factors (22).

High-risk factors	Risk Ratio for recurrence (95% CI)	Risk Ratio for metastasis (95% CI)	Risk Ratio for disease-specific-death (95% CI)
**Tumor-related (clinical)**			
Tumor diameter > 20 mm	3.22 (1.91-5.45)	6.15 (3.56-10.65)	19.10 (5.80-62.95)
Primary tumor site at:			
Temple	3.20 (1.12-9.15)	2.82 (1.72-4.63)	1.80 (0.22-14.79)
Ear	1.28 (0.56-2.90)	2.33 (1.67-3.23)	4.67 (1.28-17.12)
Lip	1.28 (0.41-3.97)	2.28 (1.54-3.37)	4.55 (1.41-14.69)
**Tumor-related (pathological)**			
Thickness > 6 mm	7.13 (3.04-16.72)	6.93 (4.02-11.94)	NR
Invasion beyond subcutaneous fat	7.61 (4.17-13.88)	11.21 (3.59-34.97)	4.49 (2.05-9.82)
Poor differentiation	2.66 (1.72-4.14)	4.98 (3.30-7.49)	5.65 (1.76-18.20)
Perineural invasion	4.30 (2.80-6.60)	2.95 (2.31-3.75)	4.06 (3.10-5.32)
**Patient-related**			
Immunosuppression	1.51 (0.81-2.81)	1.59 (1.07-2.37)	0.35 (0.05-2.58)

CI, confidence interval.

There are several available staging systems for CSCC but each of them presents some important pitfalls and may not be able to fully provide an adequate risk stratification for all cases. The American Joint Committee on Cancer (AJCC) 8th edition classification does not perform well especially regarding T stage, as few tumors fit the criteria for T4, but most T2 tumors actually turn out to be associated with poor outcomes (23). Brigham and Women’s Hospital (BWH) and the Breuninger systems are more accurate in stratifying the risk of T stage but are limited to the classification of primary tumors only (24). Finally, neither the AJCC nor the BWH staging systems consider immunosuppression, which is included as a major high-risk factor in the EADO and NCCN guidelines (20, 21, 25). Indeed, immunosuppression associated with conditions such as solid organ transplantation (26), HIV infection, and chronic lymphocytic leukemia (CLL), is not only a risk factor for increased incidence of CSCC, but also a risk factor for a more unfavorable outcome (20). Therefore, further efforts are needed to develop a dedicated classification for CSCC that could be more useful in daily clinical practice for risk stratification and early identification of high-risk CSCC (20).

Advanced CSCC is defined as a tumor for which neither surgery nor radiation therapy with curative intent is indicated (21). This broad definition is driven by the fact that there is no precise consensus on when CSCC can be considered advanced (27). In addition, contraindication to surgery or radiation therapy with curative intent may be due to several reasons which include not only the anatomic extent of the tumor, but also the patient’s clinical condition, comorbidities, the risk of mutilation or severe functional loss due to the surgery, previous treatments performed, and patient preference (27).

The advanced form can be divided into locally advanced and metastatic (loco-regional and distant). Advanced forms are considered rare; it is estimated that only about 5% of total CSCC cases may become advanced, with the limitations of missing epidemiologic data (20). Unfortunately, while the definition of metastatic CSCC (mCSCC) implies the dissemination of tumor cells through locoregional lymph nodes or both distant lymph node and other visceral sites, there are no precise parameters for defining the locally advanced forms, and a multidisciplinary discussion is essential for defining the best diagnostic and therapeutic strategies. In general, a locally advanced CSCC (laCSCC) is a tumor which is no longer eligible for either surgery or curative radiation therapy due to multiple recurrences, large extension, bone erosion and/or deep infiltration beyond the subcutaneous tissue into muscles/nerve. Moreover, the definition of laCSCC could fit tumor masses where curative resection may lead to unacceptable complications, morbidity or deformity (27). Finally, multiple CSCCs related to genetic syndromes as xeroderma pigmentosum and those related to chronic conditions such as chronic lymphocytic leukemia (CLL) may be included in these criteria (27). Patient-related features, such as age, comorbidities and patient preferences, may also play a role in the choice of either surgery or immunotherapy.

### The *Old* Therapeutic Options

Before immunotherapy, in addition to palliative radiotherapy, chemotherapy and targeted therapy with EGFR (Epidermal Growth Factor Receptor) inhibitors were the only available therapeutic options for advanced CSCC (21). In particular, chemotherapy can be considered in different treatment settings depending on the therapeutic purpose: (1) curative intent concurrent with radiation therapy, based on squamous cell carcinoma of the head and neck (HNSCC) clinical trials data. In fact, Pignon and colleagues published a meta-analysis conducted on 17,346 patients with HNSCC, demonstrating a survival benefit of concurrent chemoradiation (28). Notably, this benefit was not significant in the population over 70 years of age (28); (2) postoperative concomitant chemoradiation. A chemoradiation approach *versus* radiotherapy alone in a postoperative setting has been evaluated in a study including a population with at least one of the following high-risk features: intraparotid nodal disease, cervical nodal disease, primary tumor > 5 cm, primary tumor invading surrounding cartilage, skeletal muscle, or bone, and in-transit metastases. The study showed no differences between the two study arms in terms of either locoregional relapse or OS (29); (3) palliative intent, with questionable benefit in terms of quality of life (QoL) and overall survival (OS). Retrospective data showed that platinum derivatives appear to be the most active drugs in terms of progression-free survival (PFS) of 9.8 months and OS of 15.2 months (30). Other therapeutic options may be fluoropyrimidines (capecitabine), taxanes, bleomycin, adriamycin, and methotrexate, with PFS of approximately 5.5 months and OS of 10.9 months (30).

Regarding EGFR inhibitors, there are limited data in the curative and postoperative setting. Specifically, postoperative cetuximab concurrent with radiotherapy (n=29) *versus* radiation therapy alone (n=39) in patients with high-risk head and neck CSCC (high grade of differentiation, perineural or lymphovascular invasion, positive surgical margins, lymph node involvement, tumor recurrence, immunosuppression, localization to ear, cheek, lip), showed an advantage in terms of both freedom from local recurrence and freedom from distant recurrence (31). In the advanced setting, a phase II study including 36 patients with CSCC showed a response rate of 28% with a median duration of response of 6.8 months (32). Similar results were also observed with dacomitinib, with grade 3-4 adverse events being observed in 36% of patients, and 16% of patients discontinuing treatment because of drug-related toxicity (33). Finally, in a large retrospective case series, both chemotherapy and targeted therapy for the treatment of advanced CSCC showed response rates of less than 20%, with overall survivals of less than 20 months (34).

In summary, these treatment approaches were unsatisfactory, both in their impact on survival and quality of life (21), and a standard regimen for the treatment of advanced CSCC was not clearly defined, with up to 60% of patients with locally advanced CSCC not receiving any systemic therapy at all (35).

### The *New* Therapeutic Options

The therapeutic paradigm of CSCC has been radically changed in recent years with the introduction of immunotherapy (21). For this reason, it is crucial to discuss each advanced case in a multidisciplinary setting to properly balance the risks and benefits of this treatment in a population commonly affected by severe comorbidities and to assess the most appropriate therapeutic strategy.

Immunotherapy is considered the breakthrough in the treatment of advanced CSCC. The available clinical evidence is supported by a strong preclinical rationale. UV radiation is the most relevant risk factor for CSCC, which in fact is among the tumors with the highest rate of somatic mutations (36). The high tumor mutational burden (TMB) sets the background for a large number of neoantigens that can be recognized by the immune system. The high number of somatic mutations found in CSCC provided the strong biological rationale for the development of immunological therapies. Indeed, several studies observed that CSCC is the tumor with the highest TMB (7), with a linear relationship between tumor mutational burden and immunotherapy efficacy (37). Moreover, CSCC is a typical tumor of the elderly, with a mean age of onset of 70 years, while it is extremely rare in subjects younger than 45 years of age. Some evidence suggests that the chance of obtaining benefit from immunotherapy may increase with age. In a study involving more than 500 melanoma patients treated with PD-1 inhibitors, the risk of disease progression decreased by 13% for each decade of age (38). Finally, CSCC is characterized by high expression of Programmed Death-Ligand 1 (PD-L1) (39). The interaction of this ligand with Programmed Death-1 (PD-1) results in the inhibition of the anti-tumor immune T cell response (40). This immune checkpoint is exploited by cancer cells to escape the immune response and is one of the mechanisms underlying the rationale for the use of PD-1 inhibitors in the treatment of CSCC.

One of the first clinical evidence supporting the use of the anti-PD-1 immunotherapy for the treatment of advanced CSCC was provided by the CARSKIN trial, where first-line therapy with pembrolizumab in patients with unresectable CSCC demonstrated an objective response rate at week 15 of treatment (ORR_W15_) of 55% in PD-L1^+^ patients *versus* 17% in PD-L1^-^ patients (41). In the subsequent phase II KEYNOTE-629 trial, 105 patients with locally advanced, metastatic, or relapsed CSCC received pembrolizumab as a first-line treatment (13%) or subsequent to another systemic therapy (87%) achieving an ORR of 34% and disease control rate (DCR) of 52%. The safety profile was also acceptable and consistent with that observed in previous trials with pembrolizumab (42). As already mentioned, the results of this phase 2 trial led to the approval by the FDA of pembrolizumab for the treatment of advanced CSCC.

Before that, cemiplimab was approved by the FDA in 2018, and then by EMA, for the treatment of both mCSCC (nodal or distant metastases) and laCSCC (locally advanced) which are not eligible for curative surgery or radiation therapy, following the results of a phase 1 study that showed durable responses in 50% of 26 treated patients (18). These results were confirmed in the phase 2, open-label, non-randomized EMPOWER-CSCC 1 trial, where 193 patients with advanced, non-eligible for curative surgery or radiotherapy CSCC were enrolled. In the locally advanced CSCC group, patients were considered non-eligible for surgery if the anatomical location of the tumor would cause serious functional and aesthetic consequences (38% of cases). Other causes of inoperability were previous recurrences of the same lesion (32%) and the impossibility to obtain a complete surgical resection due to severe local invasiveness (26%). The most frequent cause of contraindication to radiotherapy was an unfavorable risk/benefit ratio (49% of cases) (18). At the last update presented at ASCO Annual Meeting 2020, the pooled ORR was 46.1% with a DCR of 72.5% (43). Clinical activity was observed regardless of PD-L1 expression (43). In addition, approximately half of patients achieved an anti-tumor response within the first 2 months, and nearly 80% within the first 4 months (43). The study showed that patients with laCSCC receiving cemiplimab after more than one recurrence after surgical excision had less than half the probability of achieving a response if compared to patients receiving upfront immunotherapy (43). This makes it essential, in the case of lesions that are potentially resectable but for which a curative outcome cannot be reasonably expected with surgery (i.e., in the presence of major risk factors), a careful multidisciplinary evaluation considering cemiplimab as a first-line treatment.

Cemiplimab has shown benefits not only in terms of clinical activity and efficacy, but also in terms of safety and quality of life. In fact, the toxicity profile of cemiplimab is comparable to that observed with other PD-1 inhibitors. Only 5% of patients had to discontinue therapy due to an adverse event of grade 3 or higher (18). According to health-related quality of life data, cemiplimab led to a clinically relevant improvement in terms of both QLQ-C30 pain scale and QLQ-C30 global health status (44).

Regarding special populations such as organ transplant patients, limited data are available. A recent systematic review showed that among 57 transplanted patients who received an immune checkpoint inhibitor for advanced malignancies, 37% experienced organ rejection, and 14% died due to rejection (45). Most of the observed rejections were among kidney (40%), liver (35%), and heart (20%) transplant patients (45). The overall response rate was 30-40% for PD-1 inhibitors (45). In case of advanced CSCC, a careful multidisciplinary approach is required to assess the risk of organ rejection and the benefit of PD-1 inhibitor treatment. In addition, patients should be fully informed of the possible risks and benefits before starting treatment with immune checkpoint inhibitors. In addition, retrospective data of 12 patients with HIV infection and advanced malignancies treated with PD-1/PD-L1 inhibitor therapy showed objective responses without unexpected adverse events nor significant impact on HIV viremia (38). In another study, pembrolizumab showed to be safe in HIV-infected patients, in particular in maintaining CD4+ T-cell count and viral suppression (46).

## Future Perspectives

The ongoing clinical studies recruiting patients with advanced or high-risk CSCC are summarized in **Table 2**. In most trials a treatment regimen including a PD-1 inhibitor is being investigated, and especially in earlier settings, such as high-risk CSCC. Most significantly, in the R2810-ONC-1788 study (NCT03969004), patients with high-risk CSCC are randomized to receive cemiplimab for 1 year *versus* placebo after surgery and adjuvant radiation therapy. The primary endpoint is disease-free survival (DFS). Cemiplimab is also being investigated in the neoadjuvant setting. Specifically, Gross and colleagues presented at the European Society of Medical Oncology (ESMO) meeting 2019 data from a phase 2 study (NCT03565783) where 20 patients with stage III/IV (M0) (AJCC 8th edition) CSCC of the head and neck received 2 doses of preoperative cemiplimab achieving a 55% of pathological complete response (pCR) and a major pathology response (MPR) in 15% (47). There were no grade ≥ 3 adverse events (47).

**Table 2:** The principal ongoing clinical studies recruiting patients with advanced or high risk CSCC.

Drug(s)	Name of clinical trial	Phase	NCT number	Status	Estimated completion date
Pembrolizumab	Neoadjuvant Study of PD-1 Inhibitor Pembrolizumab in PD-1 Naive Cutaneous Squamous Cell Carcinoma (CSCC)	2	NCT04808999	Not yet recruiting	October 2028
Atezolizumab	Neoadjuvant Atezolizumab in Surgically Resectable Advanced Cutaneous Squamous Cell Carcinoma	2	NCT04710498	Not yet recruiting	September 2024
Cemiplimab	Neoadjuvant Plus Adjuvant Treatment With Cemiplimab in Cutaneaous Squamous Cell Carcinoma	2	NCT04632433	Recruiting	February 2026
Nivolumab or Nivolumab plus Ipilimumab	Neoadjuvant Nivolumab or Nivolumab With Ipilimumab in Advanced Cutaneous Squamous Cell Carcinoma Prior to Surgery	2	NCT04620200	Recruiting	November 2024
Cemiplimab	Cemiplimab Before and After Surgery for the Treatment of High Risk Cutaneous Squamous Cell Cancer	1	NCT04428671	Recruiting	October 2030
Cemiplimab (47)	Cemiplimab in Treating Participants With Recurrent Stage III-IV Head and Neck Squamous Cell Cancer Before Surgery	2	NCT03565783	Recruiting	July 2021
Cemiplimab	Cemiplimab in AlloSCT/SOT Recipients With CSCC	1	NCT04339062	Recruiting	July 2022
Cemiplimab	A PD-1 Checkpoint Inhibitor (Cemiplimab) for High-Risk Localized, Locally Recurrent, or Regionally Advanced Skin Cancer	2	NCT04315701	Recruiting	January 2023
Nivolumab	Nivolumab for Treatment of Squamous Cell Carcinoma of the Skin	2	NCT04204837	Active, not recruiting	December 2023
Talimogene Laherparepvec and Panitumumab	Talimogene Laherparepvec and Panitumumab for the Treatment of Locally Advanced or Metastatic Squamous Cell Carcinoma of the Skin	1	NCT04163952	Recruiting	September 2024
IFx-Hu2.0 Vaccine	Immunotherapy With IFx-Hu2.0 Vaccine for Advanced MCC or CSCC	1	NCT04160065	Recruiting	June 2022
Cemiplimab	Study of Cemiplimab in Patients With Type of Skin Cancer Stage II to IV Cutaneous Squamous Cell Carcinoma	2	NCT04154943	Recruiting	December 2024
Cemiplimab with and without RP1	Study Evaluating Cemiplimab Alone and Combined With RP1 in Treating Advanced Squamous Skin Cancer	2	NCT04050436	Recruiting	March 2025
Cemiplimab	Study of Adjuvant Cemiplimab Versus Placebo After Surgery and Radiation Therapy in Patients With High Risk Cutaneous Squamous Cell Carcinoma	3	NCT03969004	Recruiting	February 2027
Avelumab with or without Cetuximab	Avelumab With or Without Cetuximab in Treating Patients With Advanced Skin Squamous Cell Cancer	2	NCT03944941	Recruiting	December 2023
Intralesional Cemiplimab	Pre-Operative Cemiplimab Administered Intralesionally for Patients With Recurrent Cutaneous Squamous Cell Carcinoma	1	NCT03889912	Active, not recruiting	February 2022
Nivolumab	Nivolumab in Patients With Advanced Cutaneous Squamous Cell Carcinoma	2	NCT03834233	Active, not recruiting	December 2022
Pembrolizumab	Pembrolizumab Versus Placebo Following Surgery and Radiation in Participants With Locally Advanced Cutaneous Squamous Cell Carcinoma (MK-3475-630/KEYNOTE-630)	3	NCT03833167	Recruiting	September 2028
Avelumab plus Radiotherapy	The UNSCARRed Study: UNresctable Squamous Cell Carcinoma Treated With Avelumab and Radical Radiotherapy	2	NCT03737721	Recruiting	June 2023
Intratumoral Cavrotolimod With Pembrolizumab or Cemiplimab	Intratumoral Cavrotolimod Combined With Pembrolizumab or Cemiplimab in Patients With Merkel Cell Carcinoma, Cutaneous Squamous Cell Carcinoma, or Other Advanced Solid Tumors	1/2	NCT03684785	Recruiting	June 2023
Lenvatinib plus Cetuximab	Testing Lenvatinib and Cetuximab in Patients With Advanced Head and Neck Squamous Cell Carcinoma and Cutaneous Squamous Cell Carcinoma	1	NCT03524326	Recruiting	April 2023
Pembrolizumab with or without Cetuximab	Immunotherapy +/- EGFR Inhibitor In Advanced/Metastatic cSCC: Tackling Primary And Secondary Resistance (I-Tackle)	2	NCT03666325	Not yet recruiting	October 2022

AlloSCT/SOT, allogenic stem cell transplantation/solid organ transplantation; CSCC, cutaneous squamous cell carcinoma; MCC, merkel cell carcinoma; PD-1, Programmed Death-1.

Immunotherapy has led to pivotal changes in advanced CSCC both in terms of objective responses, survival, and improved quality of life. However, patients with advanced CSCC receiving immunotherapy after more than one recurrence after surgical excision had less than half the probability of achieving an objective response (43). This could be related to primary or secondary resistance to immunotherapy (48). For this reason, clinical trials are ongoing with the aim of overcoming resistance to immunotherapy. In fact, the combination of PD-1 or PD-L1 inhibitors with other agents (such as radiotherapy, oncolytic viruses, or EGFR inhibitors) is being investigated to overcome primary or secondary resistance to immunotherapy, such as in the I-Tackle trial (NCT03666325) with the addition of cetuximab to pembrolizumab at primary or acquired resistance; or in the UNSCARRed study (NCT03737721) with the addition of radiotherapy to avelumab.

## Discussion and Conclusions

**Figure 1:**
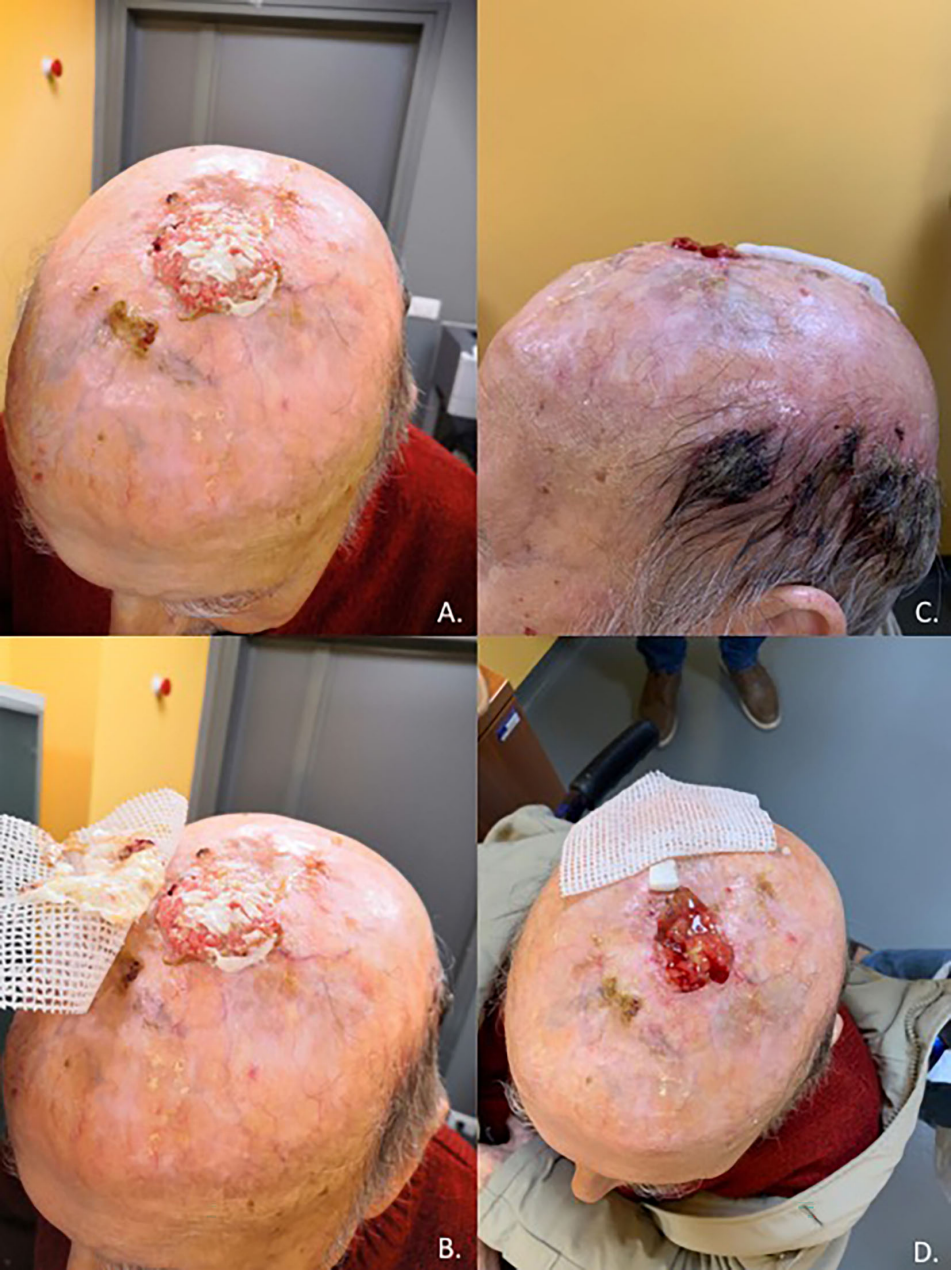
Case report of a 92-year-old man with unresectable, non-eligible to curative radiotherapy, locally advanced CSCC invading the skullcap and leptomeningeal membrane **(A, B)** who achieved a rapid clinical response after one cycle of Cemiplimab **(C, D)**.

**Figure 2:**
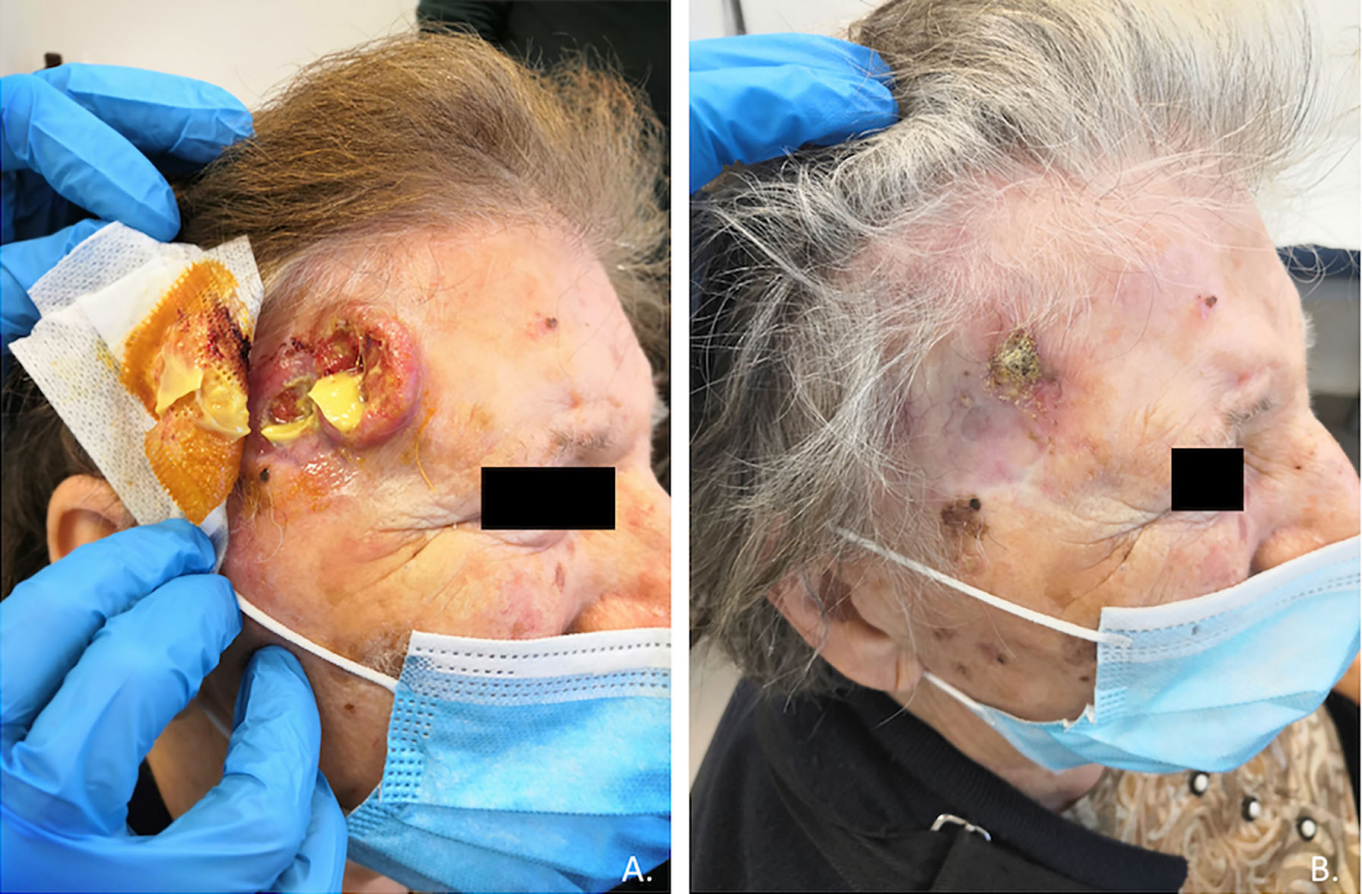
Case report of a rapid clinical response, after only one course of therapy with cemiplimab, in an 83-year-old patient with locally advanced recurrence of cutaneous squamous carcinoma of the right temporal region **(A,B)**.

Cutaneous squamous cell carcinoma is a common condition, although it remains rare in its advanced stages; high-risk cases require multidisciplinary care due to the complexity associated with both the disease and the often frail population (27). Before the introduction of immunotherapy in clinical practice, a standard of care for advanced CSCC was not clearly defined, and up to 60% of patients with advanced CSCC did not receive any systemic therapy at all, due to the low clinical activity and high risk of severe toxicities (21). Based on a strong preclinical rationale, clinical trials were conducted leading to the registration by the regulatory authorities of anti-PD-1 immunotherapy in patients with advanced CSCC (21). Cemiplimab was the first PD-1 inhibitor receiving an indication in CSCC after showing in a clinical trial rapid and durable responses in more than 40% of patients (in **Figures 1**, **2** we reported two clinical cases of rapid clinical response), with a favorable safety profile. In addition to that, cemiplimab led to an improvement in health-related quality of life with a reduction in cancer-related pain after a few cycles of therapy (18, 43, 44).

Anti-PD-1 drugs are the backbone of current clinical investigation in patients with CSCC. Specifically, several clinical trials with PD-1 inhibitors are currently underway investigating the activity, efficacy, and safety of adjuvant approaches in individuals with high-risk CSCC, and neoadjuvant approaches in patients with advanced CSCC. Based on the results of these studies, anti-PD-1 drugs may soon become standard of care in the adjuvant and neoadjuvant settings.

## Author Contributions

FS and PQ jointly supervised this work. All authors contributed to the article and approved the submitted version.

## Conflict of Interest

FS received honoraria for presentations or lectures from Sanofi, Roche, BMS, Novartis, Merk, SunPharma, MSD, Pierre Fabre, and surved on advisory boards of Novartis, Philogen, SunPharma and MSD; PQ reports consulting or advisory role for Bristol Myers Squibb, Merck & Co., Novartis, Pierre Favre, Roche/Genentech, and Sanofi.

The remaining authors declare that the research was conducted in the absence of any commercial or financial relationships that could be construed as a potential conflict of interest.

## Publisher’s Note

All claims expressed in this article are solely those of the authors and do not necessarily represent those of their affiliated organizations, or those of the publisher, the editors and the reviewers. Any product that may be evaluated in this article, or claim that may be made by its manufacturer, is not guaranteed or endorsed by the publisher.
